# Aldehyde Dehydrogenase 1 Identifies Cells with Cancer Stem Cell-Like Properties in a Human Renal Cell Carcinoma Cell Line

**DOI:** 10.1371/journal.pone.0075463

**Published:** 2013-10-08

**Authors:** Kosuke Ueda, Sachiko Ogasawara, Jun Akiba, Masamichi Nakayama, Keita Todoroki, Keiko Ueda, Sakiko Sanada, Shigetaka Suekane, Masanori Noguchi, Kei Matsuoka, Hirohisa Yano

**Affiliations:** 1 Department of Pathology, Kurume University School of Medicine, Kurume, Fukuoka, Japan; 2 Department of Urology, Kurume University School of Medicine, Kurume, Fukuoka, Japan; 3 Research Center of Innovative Cancer Therapy, Kurume University School of Medicine, Kurume, Fukuoka, Japan; Okayama University, Japan

## Abstract

Cancer stem cells (CSC) or cancer stem cell-like cells (CSC-LCs) have been identified in many malignant tumors. CSCs are proposed to be related with drug resistance, tumor recurrence, and metastasis and are considered as a new target for cancer treatment; however, there are only a few reports on CSCs or CSC-LCs in renal cell carcinoma (RCC). Different approaches have been reported for CSC identification, but there are no universal markers for CSC. We used two different approaches, the traditional side population (SP) approach, and the enzymatic (aldehyde dehydrogenase 1 (ALDH1)) approach to identify CSC-LC population in two RCC cell lines, ACHN and KRC/Y. We found that ACHN and KRC/Y contain 1.4% and 1.7% SP cells, respectively. ACHN SP cells showed a higher sphere forming ability, drug resistance, and a slightly higher tumorigenic ability in NOD/SCID mice than Non-SP (NSP) cells, suggesting that cells with CSC-LC properties are included in ACHN SP cells. KRC/Y SP and NSP cells showed no difference in such properties. ALDH1 activity analysis revealed that ACHN SP cells expressed a higher level of activity than NSP cells (SP vs. NSP: 32.7% vs 14.6%). Analysis of ALDH1-positive ACHN cells revealed that they have a higher sphere forming ability, self-renewal ability, tumorigenicity and express higher mRNA levels of CSC-LC property-related genes (e.g., ABC transporter genes, self-replication genes, anti-apoptosis genes, and so forth) than ALDH1-negative cells. Drug treatment or exposure to hypoxic condition induced a 2- to 3-fold increase in number of ALDH1-positive cells. In conclusion, the results suggest that the ALDH1-positive cell population rather than SP cells show CSC-LC properties in a RCC cell line, ACHN.

## Introduction

Renal cell carcinoma (RCC) is one of the most common malignancies of the genitourinary tract, accounting for 116,500 deaths in 2008 according to the World Health Organization [1]. The incidence of RCC has been steadily rising over the past 30 years [2]. Furthermore, because metastatic RCC is notoriously resistant to most conventional therapies, such as chemotherapy and radiotherapy, the prognosis of patients with RCC is poor as one-third of patients already have metastatic disease at the initial diagnosis and 30–40% of them develop distant metastases after resection of the primary tumor [3]. In recent years, the molecular targeted therapies that have been developed have shown significant objective responses [4]–[6], and they are now recognized as the current standard therapies of metastatic RCC. However, the efficacy of these molecular target therapies is insufficient.

The two dominant models of carcinogenesis are the stochastic model (clonal evolution) and the hierarchic organization of tumor (cancer stem cell (CSC)) model. According to the traditional clonal evolution model, tumor formation is the consequence of accumulating random genetic events in normal differentiated cells, whereas the CSC model postulates that a single CSC gives rise to a hierarchical organization within a tumor [7], [8]. Recent studies suggest that CSCs may be responsible for tumorigenesis and contribute to some individuals’ resistance to cancer therapy, which resulted in cancer relapse and metastasis [9], [10]. Therefore, it is widely believed that identification and characterization of CSC or cancer stem cell-like cell (CSC-LC) may contribute significantly to the development of effective therapies. Bussolati et al. identified a population of CD105 positive tumor initiating cells in RCCs, and reviewed the literature on the role of stem cells in human RCC [11], [12]. Kim et al. reported that the expression of stem cell markers, OCT4 and CD133, may serve, respectively, as a poor and favorable prognostic marker, in papillary RCC [13]. In addition, they suggested that the expression of CD133 is a favorable prognostic marker in clear cell RCC [14].

There are many reports that CSC-LCs of some solid tumors are present in side population (SP) cells [15], [16], but there are only a few reports on the role of SP cells in human RCC [17], [18]. SP cells were originally identified in flow cytometric analyses by their ability to efflux the vital DNA dye, Hoechst 33342, resulting in Hoechst-negative SP cells and Hoechst-positive Non-SP (NSP) cells. Previous studies of cancers in vitro and primary tumors in vivo have shown that SP cells are uniquely capable of generating both SP and NSP cell populations, exhibiting properties consistent with stem cells or CSC. SP cells express high levels of ATP-binding cassette (ABC) transporter family members, especially ABCG2, and exhibit more chemotherapeutic drug resistance than NSP cells in cell lines derived from some human malignant solid tumors, such as breast cancer, lung cancer, ovarian cancer and squamous cell cancer [19]–[21].

Recently, it has been reported that aldehyde dehydrogenase 1 (ALDH1) is responsible for the oxidation of retinol to retinoic acid and plays pivotal roles in embryonic development and homeostasis in several organs [22]. Some researchers have reported that high expression of ALDH1 was associated with drug resistance and poor prognosis, and that ALDH1 is a CSC marker [23], [24]. Ozbek et al. reported that ALDH1 expression was correlated with tumor grade in RCC [25], but the biological features of ALDH1-positive cells in RCC are still largely unknown.

In this study, we isolated SP cells from two human RCC cell lines and systematically investigated the CSC properties of the SP cells and ALDH1-positive cells, and relationship between SP cells and ALDH1-positive cells.

## Materials and Methods

### Cell Lines and Animals

We used two RCC cell lines: one derived from malignant pleural effusion of a patient with RCC (ACHN) and the other derived from primary lesion of a patient with RCC (KRC/Y). These 2 RCC cell lines have high proliferative and colony forming abilities *in vitro* and possess high tumorigenicity in even nude mice *in vivo*. ACHN was purchased from American Type Culture Collection. KRC/Y was established in our laboratory [26]. Culture medium for ACHN consisted of modified Eagle’s medium (EMEM) (Gibco, BRL/Life Technologies Inc., Gaithersburg, MD, USA). Culture medium for KRC/Y consisted of Dulbecco’s modified medium (DMEM) (Nissui Seiyaku Co., Tokyo, Japan) supplemented with heat-inactivated (56°C, 30 min) 5% fetal bovine serum (FBS, Bioserum, Vic, Australia), 100 U/mL penicillin and 100 µg streptomycin (Gibco BRL/Life Technologies Inc.). Cells were cultured in an atmosphere of 5% CO_2_ in air at 37°C. Female non-obese diabetic/severe combined immunodeficiency (NOD/SCID) mice (5 week-old) were purchased (Clea Japan, Inc., Osaka, Japan), and housed in laminar-flow cabinets under specific pathogen-free conditions. All procedures were approved by the Ethics Review Committee for Animal Experimentation of Kurume University School of Medicine.

### Expression of CSC Markers in RCC Cell Lines

We analyzed the expression of the putative CSC markers ABCG2, CD90, CD105, CD133 and epithelial cell adhesion molecule (EpCAM) in ACHN and KRC/Y. Cells were incubated in the dark at 4°C for 30 minutes with fluorescence-conjugated monoclonal antibodies, including fluorescein isothiocyanate (FITC)-conjugated mouse anti-human CD90 antibody (5E10, BD Biosciences, San Jose, CA, USA) and mouse anti-human CD105 antibody (MEM-226, EXBIO, Praha, Czech) and phycoerythrin (PE)-conjugated CD133/2 antibodies (293C3, Miltenyi Biotec, Bergisch-Gladbach, Germany) and anti-EpCAM antibody (EBA-1, BD Biosciences). Cells with mouse anti-BCRP monoclonal antibody (ABCG2) (BXP-21, Chemicon, Temecula, CA, USA) were incubated for 30 minutes and further incubated in the dark at 4°C for 30 minutes with FITC-conjugated goat anti-mouse Ig (FITC-GAM) (BD Biosciences). Cells were washed, resuspended and analyzed on a FACScan (Becton Dickinson, Franklin Lakes, NJ, USA).

### SP Cell Identification and CSC Marker Expression in SP and NSP Cells

Cultured cells with 80% confluence were detached with accutase (Innovative Cell Technologies, Inc., San Diego, USA) and suspended at 1×10^6^ cells/mL in phosphate-buffered saline (PBS) supplemented with 2% FBS and then incubated with Hoechest 33342 dye alone (5 µg/mL for ACHN and 10 µg/mL for KRC/Y) (SIGMA-Aldrich, Saint Louis, MO, USA) or with 20 µg/mL reserpine (SIGMA-Aldrich) at 37°C for 60 min. Samples were washed, centrifuged and resuspended in 2 mL cold PBS supplemented with 2% FBS, then 1 µg/mL propidium iodide (PI) (BD Biosciences) was added and the cells were filtered through a 40 µm cell strainer (BD Biosciences). Flow cytometric analysis was performed as previously described [27]. Reserpine is conventionally used as a guiding parameter to determine the boundary between SP and NSP cells. Analyses were carried out with a FACSAria II (BD Biosciences). The expression of CD90 and EpCAM in ACHN, and that of CD105 and EpCAM in KRC/Y, in SP and NSP cells was further examined. Cells were stained using the method described above.

### Cell Growth Assay of SP and NSP Cells

A total of 2,000 SP cells and NSP cells were plated in 96-well plates and cultured in a CO_2_ incubator. The cells were harvested at 24, 48, 72, 96, 120 or 144 hours and the proliferation was examined in colorimetric assays using 3-(4,5-dimethylthiazol-2yl-yl-)-2,5-diphenyl tetrazolium bromide (MTT) cell growth assay kits (Chemicon, Temecula, CA, USA) as described elsewhere [28].

### Colony Formation Assay of SP and NSP Cells

The soft agar anchorage independent clonogenic growth assay was performed. Briefly, 2×10^4^ cells were suspended in 2 mL of EMEM or DMEM containing 0.36% soft agar (Gibco BRL/Life Technologies Inc.) and 10% FBS in a 35 mm dish. The cell suspension was then overlaid on a presolidified 0.72% hard agar. The medium containing 0.36% soft agar was supplemented once a week. Colonies (>10 cells) that arose within 3 weeks were presented as clonogenicity. Five dishes were examined for each cell type and blindly counted under the microscope (×200) in all fields.

### Sphere Formation Assay of SP and NSP Cells

Isolated SP and NSP cells from the two cell lines (4,000 cells/dish) were cultured in serum-free medium including 10 ng/mL epidermal growth factor (EGF) (Sankojunyaku, Tokyo, Japan) and 20 ng/mL basic fibroblast growth factor (bFGF) (Sankojunyaku) using ultra-low-attachment 6-well plates (Corning Inc., Corning, NY, USA) for 1 week, after which sphere formation was assessed by counting the number of spheres (>3 cells) under microscope (×200).

### Drug Resistance Assay

Isolated SP and NSP cells were planted at 2,000 cells per well in 96-well plates, and the effect of the multikinase inhibitor Sorafenib (2 µM) (Cell Signaling Technology. Inc., Danvers, MA, USA) and IFNα (4,000 IU/mL) (OIF, Otsuka Pharma Co., Ltd., Tokyo, Japan) was examined. Drug resistance was determined after treatment for 72, 96 or 144 hours by MTT assay.

### Tumorigenicity Assays of SP and NSP Cells in vivo

To explore tumorigenic capacity, SP and NSP cells (1, 10 or 100×10^3^) were isolated from the two RCC cell lines, placed in 100 µL medium, and separately injected into the subcutaneous space in the flank of five-week old female NOD/SCID mice under anaesthetization. Tumorigenic capacity was judged 8 weeks after injection.

### cDNA Preparation and Quantitative Real-time RT-PCR for Gene Expression Assay

After SP and NSP cells in ACHN and KRC/Y were isolated, total RNA was extracted using an RNeasy Plus Micro Kit (Qiagen, Valencia, CA, USA), and complementary DNA (cDNA) was synthesized using the Reverse Transcription System (Promega, Madison, WI, USA) according to the manufacturer’s instructions. Quantitative real-time RT-PCR (qRT-PCR) was performed to examine the expression of CSC-LC property-related genes (e.g., ABC transporter genes (ABCB1 and ABCG2), self-replication genes (BMI1 and c-MYC), anti-apoptosis genes (BCL2 and CFLAR), hypoxia-related genes (hypoxia inducible factor 1α (HIF1α) and vascular endothelial growth factor-A (VEGFA)), and epithelial-mesenchymal transition (EMT)-related genes (Snail and Twist)) with an ABI PRISM 7500 (Applied Biosystems, Foster City, CA, USA). Gene expression assays and primer and probe mixes were used for ABCB1, ABCG2, ALDH1A1, BMI1, c-MYC, BCL2, CFLAR, HIF1α, VEGFA, Snail, Twist, and β-actin (assay IDs (Hs 00184500_m1, Hs00184979_ml, Hs00946916_m1, Hs00180411_ml, Hs00153408_ml, Hs00608023_m1, Hs00153439_m1, Hs00153153_ml, Hs00900055_ml, Hs00195591_m1, Hs01675818_s1, and Hs99999903_m1, respectively; Applied Biosystems),and thermal cycle conditions were as follows: initial incubation at 95°C for 10 min, then 40 cycles alternating in turn with 95°C for 10 s, 60°C for 20 s, and 72°C for 15 s, and then maintained at 72°C for 10 min.

Comparative gene expression analysis was performed using the 2^(−ΔΔCt)^ methods with normalization to the level of internal control gene, β-actin.

### ALDH1 Expression in SP and NSP Cells and in Cells under Pathologic Conditions

ALDH1 expression was investigated in samples prepared from SP and NSP cells, drug-treated cells, and cells cultured under hypoxic conditions. Briefly, SP and NSP cells were isolated from ACHN and KRC/Y cells cultured for 72 hours using the method described above. Parental cells and isolated SP and NSP cells were used as samples. ACHN cells cultured with EMEM containing Sorafenib (1 µM) or IFNα (4,000 IU/m) for 48, 72 or 96 hours and the cells cultured under hypoxic (1% O_2_) conditions for 48, 72 or 96 hours were also used as samples. Samples were suspended in ALDEFLUOR assay buffer containing ALDH substrate, BAAA (Bodipy-aminoacetaldehyde) (50 µg dry reagent), with or without 5 µl of the specific ALDH inhibitor diethylaminobenzaldehyde (DEAB 1.5 mM in 95% ethanol stock solution), as a negative control, and incubated for 60 min at 37°C (ALDEFLOUR KIT, Stem cells technologies, Vancouver, BC, Canada), and analyzed using flow cytometry (FCM).

### Biologic Characteristics of ALDH1-positive and ALDH1-negative Cells

Sphere formation assay was performed in ACHN and KRC/Y cells. Tumorigenicity assay and gene expression assay were performed to examine biological features of ALDH1-positive and ALDH1-negative ACHN cells. To compare the self-renewal capacity between ALDH1-positive and ALDH1-negative ACHN cells, we examined a sphere-forming ability by three consecutive serial passages of single-dissociated cells according to the method of Lim et al. [29]. Briefly, after dissociating the first passage sphere with 0.25% trypsin, single-dissociated cells in ALDH1-positive and ALDH1-negative cells of ACHN were plated in 6-well plates. One week later, the number of spheres was counted and the same procedure was repeated once again. Tumorigenicity assay and gene expression assay were performed as described above except the comparison at 1×10^3^ cells was not performed in the tumorigenicity assay.

### Statistical Analysis

Comparison of cell growth assay was performed using two-factor factorial ANOVA, and those of colony formation assay, sphere formation assay, and drug resistance assay were performed using Student’s *t*-test. The other data comparisons were performed using the Mann-Whitney U test. A value of *P*<0.05 was considered significant.

## Results

### Expression of CSC Markers

ACHN expressed CD90 (96.9%) and EpCAM (87.7%), but expression of CD105 (1.5%), CD133 (1.3%) and ABCG2 (0.9%) remained at very low levels. On the other hand, KRC/Y expressed CD105 (28.9%) and EpCAM (93.0%), but expression of CD90 (1.7%), CD133 (1.7%), and ABCG2 (2.9%) was very low.

### SP Cells Analysis and Expression of CSC Markers in SP and NSP Cells

**Figure 1:**
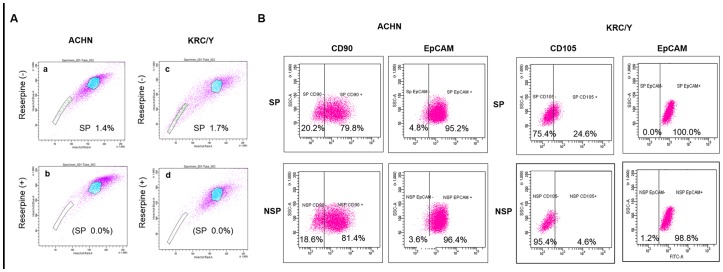
SP cells analysis and expression of CSC markers in SP and NSP cells. (A) ACHN and KRC/Y were labeled with Hoechst 33342, and then analyzed by FCM. The SP cell rates in ACHN and KRC/Y were 1.4% (A–a) and 1.7% (A–c), respectively, which decreased significantly in the presence of reserpine (A–b, A–d). The experiment was repeated at least three times for each cell line and almost identical results were obtained. A representative figure of our experiments is shown. (B) There was no apparent difference in CD90 and EpCAM expression between SP and NSP cells in ACHN. In the KRC/Y cell line, although there was no difference in EpCAM expression, SP cells expressed a higher CD105-positive cell rate than NSP cells (SP vs NSP : 24.6% vs 4.6%). The experiments were repeated twice, and almost identical results were obtained. A representative figure of our experiments is shown.

The SP cell fractions in ACHN and KRC/Y were 1.4% and 1.7%, respectively (Fig. 1A). Subsequently, we examined the expression of CSC markers, such as CD90 and EpCAM in ACHN, and CD105 and EpCAM in KRC/Y, in SP and NSP cells. There was no apparent difference in CD90 and EpCAM expression between SP and NSP cells in ACHN. Although there was no difference in EpCAM expression between SP and NSP cells in KRC/Y, CD105 expression in SP cells (24.6%) was much higher than in NSP cells (4.6%) (Fig. 1B).

### Biological Features of SP and NSP Cells in ACHN and KRC/Y in vitro

**Figure 2:**
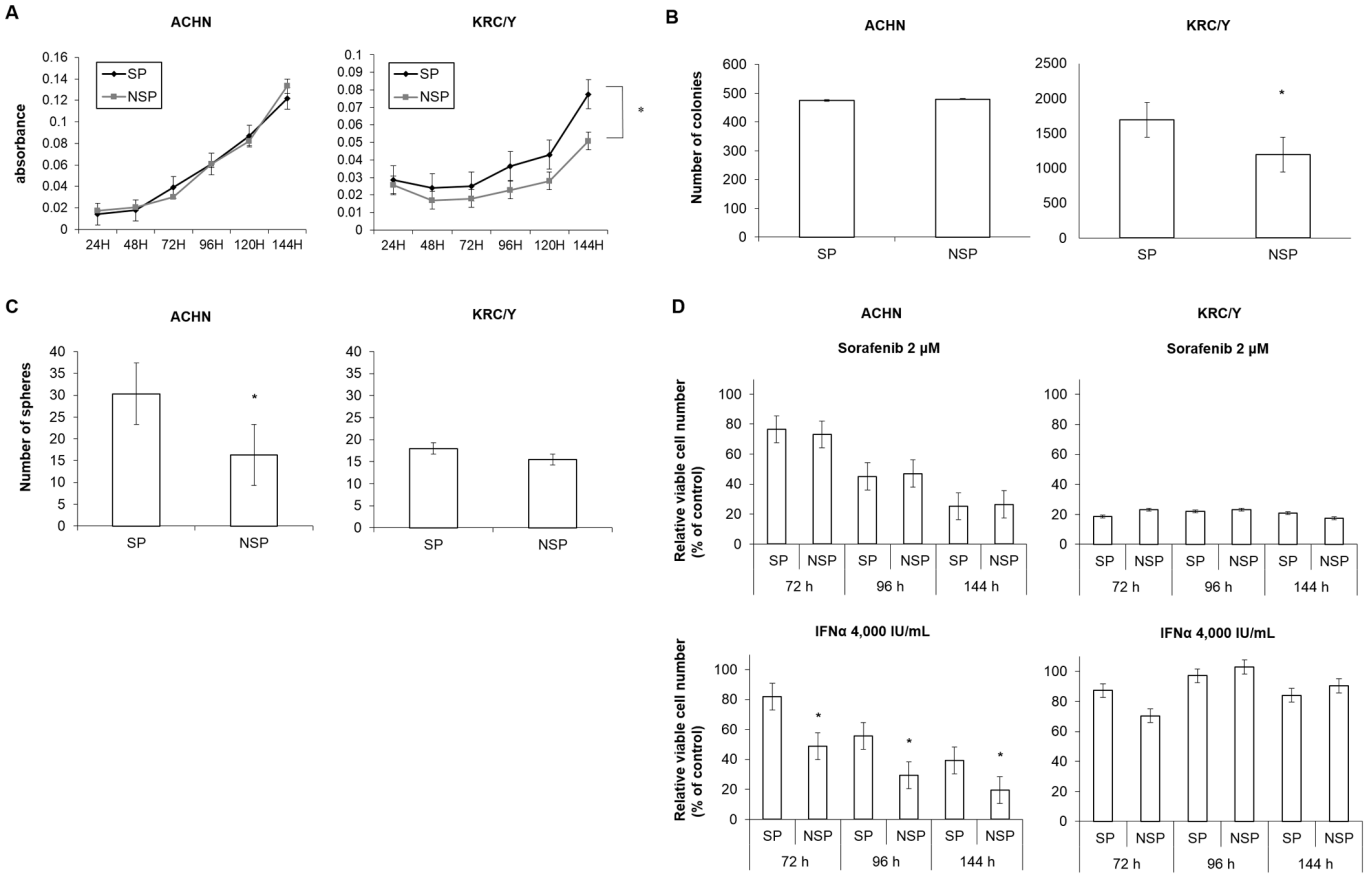
Biological features of SP and NSP cells in ACHN and KRC/Y in vitro. (A) Growth curves of SP and NSP cells. SP cells in KRC/Y showed a higher proliferative ability compared to NSP cells (* *P*<0.0001). (B) The clonogenity was significantly increased in SP cells in KRC/Y (* *P*<0.01). (C) Sphere forming ability was significantly higher in SP cells in ACHN (* *P*<0.05). (D) Drug resistance of SP and NSP cells treated with Sorafenib or IFNα. SP cells in ACHN had higher IFNα resistance (* *P*<0.0001). The experiments were repeated twice, and almost identical results were obtained.

There was no significant difference in the cell proliferative ability and clonogenicity between SP and NSP cells in ACHN. On the other hand, after culturing for 48 hours, SP cells in KRC/Y had a significantly higher proliferative ability than NSP cells (*P*<0.0001) (Fig. 2A). Although SP cells in KRC/Y had a significantly higher clonogenicity than NSP cells (*P*<0.01) (Fig. 2B), there was no significant difference in sphere forming ability between SP and NSP cells in KRC/Y. Conversely, SP cells in ACHN had a significantly higher sphere forming ability than NSP cells (Fig. 2C). After 72, 96 or 144 hours treatment with Sorafenib or IFNα, the sensitivity to each drug was assessed with the MTT assay. There was no difference in sensitivity between SP cells and NSP cells in KRC/Y against Sorafenib or IFNα treatment. However, the SP cells in ACHN had a significantly higher IFNα resistance (*P*<0.0001) (Fig. 2D).

### Tumorigenicity Assays in vivo in SP and NSP Cells

Both SP and NSP cells showed tumor forming ability in each of the two RCC cell lines. The ratio of tumorigenicity between SP and NSP cells in ACHN and KRC/Y was not significantly different, but the tumorigenicity of SP cells was slightly higher than that of NSP cells in ACHN (Table 1).

**Table 1:** Tumorigenicity of side population (SP) and Non-SP (NSP) cells in ACHN and KRC/Y.

	Injected cell number			
		1×10^3^	1×10^4^	1×10^5^
**ACHN**	**SP**	0/5	1/5	3/5
	**NSP**	0/5	0/5	1/5
**KRC/Y**	**SP**	0/5	0/5	3/5
	**NSP**	0/5	0/5	2/5

### Analysis of CSC-LC Property-related Gene Expression in SP and NSP Cells by qRT-PCR

**Figure 3:**
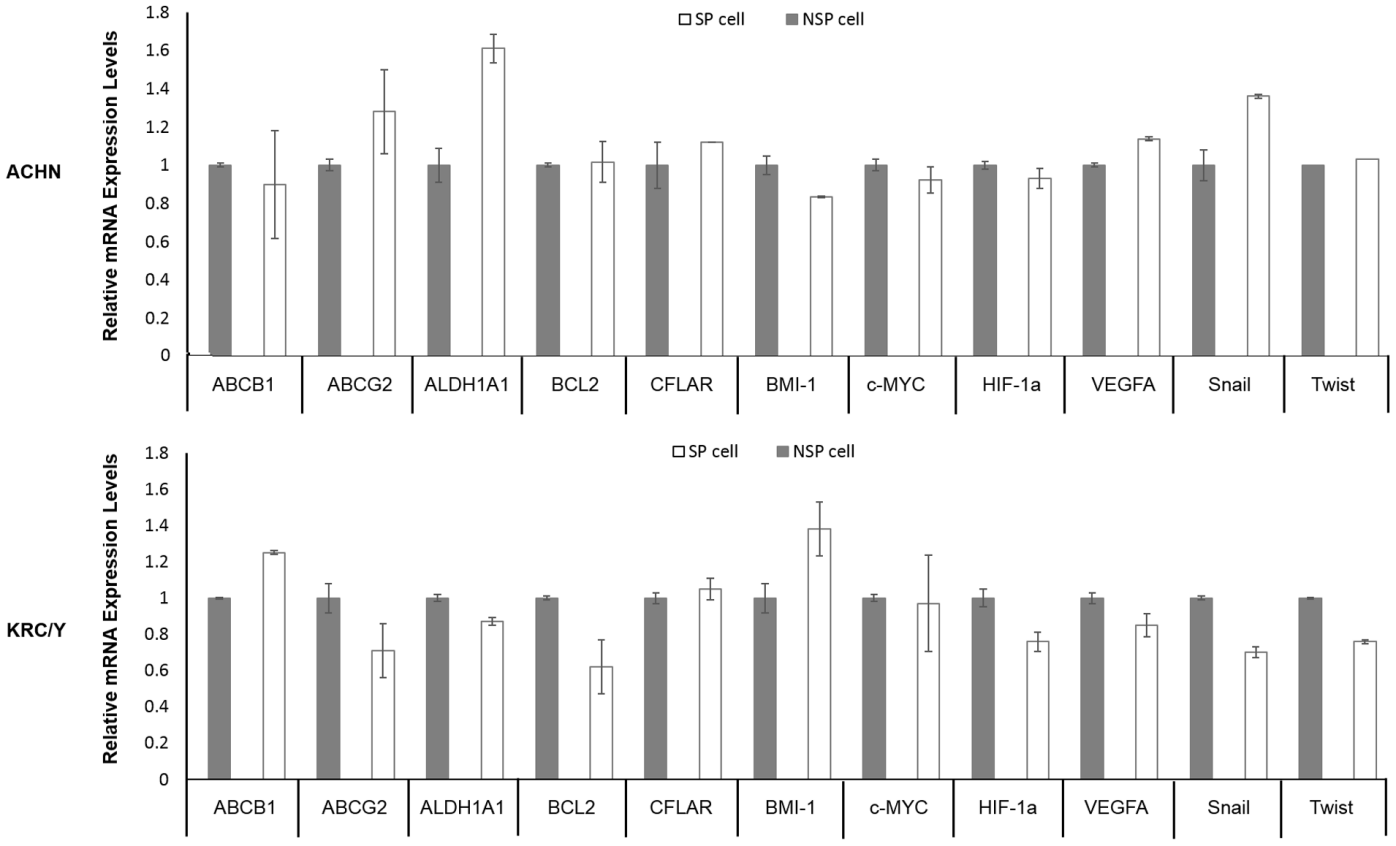
Quantification of mRNA expression of CSC-LC property-related genes in SP and NSP cells by real-time PCR. There were no significant differences in mRNA expressions of ABC transporter genes (ABCB1 and ABCG2), self-replication genes (BMI-1 and c-MYC), anti-apoptosis genes (BCL2 and CFLAR), hypoxia-related genes (VEGFA and HIF1α), and EMT-related genes (Snail and Twist) between SP and NSP cells in the 2 cell lines. SP cells in ACHN expressed a slightly higher level of ALDH1A1 mRNA than NSP cells, but no apparent difference was observed in KRC/Y. The experiment was repeated at least four times for each cell line and almost identical results were obtained.

There were no significant differences in mRNA expressions of ABC transporter genes (ABCB1 and ABCG2), self-replication genes (BMI-1 and c-MYC), anti-apoptosis genes (BCL2 and CFLAR), hypoxia-related genes (VEGFA and HIF1α), and EMT-related genes (Snail and Twist) between SP and NSP cells in the 2 cell lines. SP cells in ACHN expressed a slightly higher level of ALDH1A1 mRNA than NSP cells, but no apparent difference was observed in KRC/Y (Fig. 3).

### ALDH1 Expression, and Biological Features of ALDH1-positive and ALDH1-negative RCC Cells

**Figure 4:**
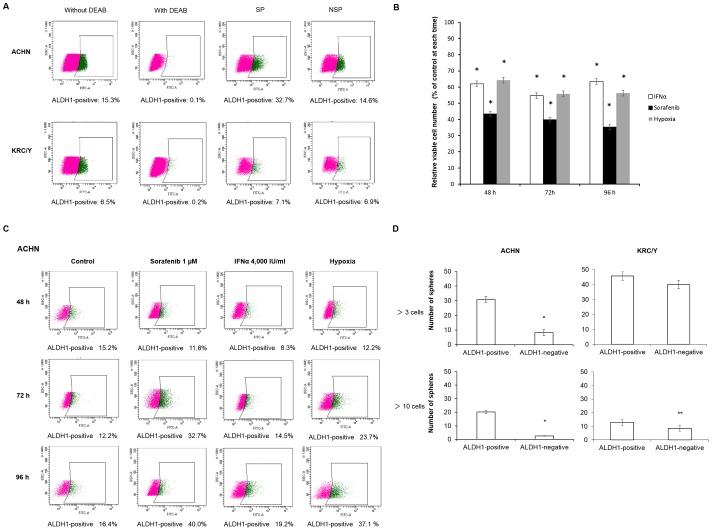
ALDH1 expression, and biological features of ALDH1-positive and ALDH1-negative RCC cells. (A) The expression of ALDH1 in SP cells and NSP cells in ACHN and KRC/Y. The ALDH1-positive cell rates in ACHN and KRC/Y were 15.3% and 6.5%, respectively. (B) Comparison of cell growth among control cells, cells treated with Sorafenib or IFNα, and cells exposed to hypoxia in ACHN. Cell growth was measured at 48, 72 or 96 hours after drug treatment or exposure to hypoxia. Cell growth after drug treatment or exposure to hypoxia was significantly suppressed as compared with control (* *P*<0.005, ** *P*<0.0001 vs. control). (C) The percentage of ALDH1-positive cells in cells treated with Sorafenib or IFNα, or cells exposed to hypoxia for 48, 72 or 96 hours. The percentage of ALDH1-positive cells in cells treated with Sorafenib or IFNα, or cells exposed to hypoxia for 96 hours was higher as compared with the normal condition. The experiments were repeated twice, and almost identical results were obtained. A representative figure of our experiments is shown. (D) Sphere forming ability between ALDH1-positive cells and ALDH1-negative cells. The sphere formation of ALDH1-positive cells in ACHN and KRC/Y was higher than that of ALDH1-negative cells. The experiments were repeated twice, and almost identical results were obtained.

The ALDH1-positive cell rate in KRC/Y cells was 6.5%. There was no difference in ALDH1 expression between SP and NSP cells. In ACHN cells, the ALDH1-positive cell rate was 15.3%. Also, the number of ALDH1-positive SP cells (32.7%) was higher than that of NSP cells (14.6%) (Fig. 4A). Cell growth was significantly suppressed in cells treated with Sorafenib or IFNα and in cells exposed to hypoxia, as compared with control cells (Fig. 4B). Regarding ALDH1 expression, there was no apparent difference in ALDH1-positive cell rates among control cells, cells treated with Sorafenib or IFNα, and cells exposed to hypoxic condition for 48 hours. However, the percentage of ALDH1-positive cells increased chronologically, especially in cells treated with Sorafenib or exposed to hypoxic conditions. In particular, after exposure to Sorafenib or IFNα, or hypoxia for 96 hours, the percentages of ALDH1-positive cells were 40.0%, 19.2% and 37.1%, respectively (Fig. 4C).

**Figure 5:**
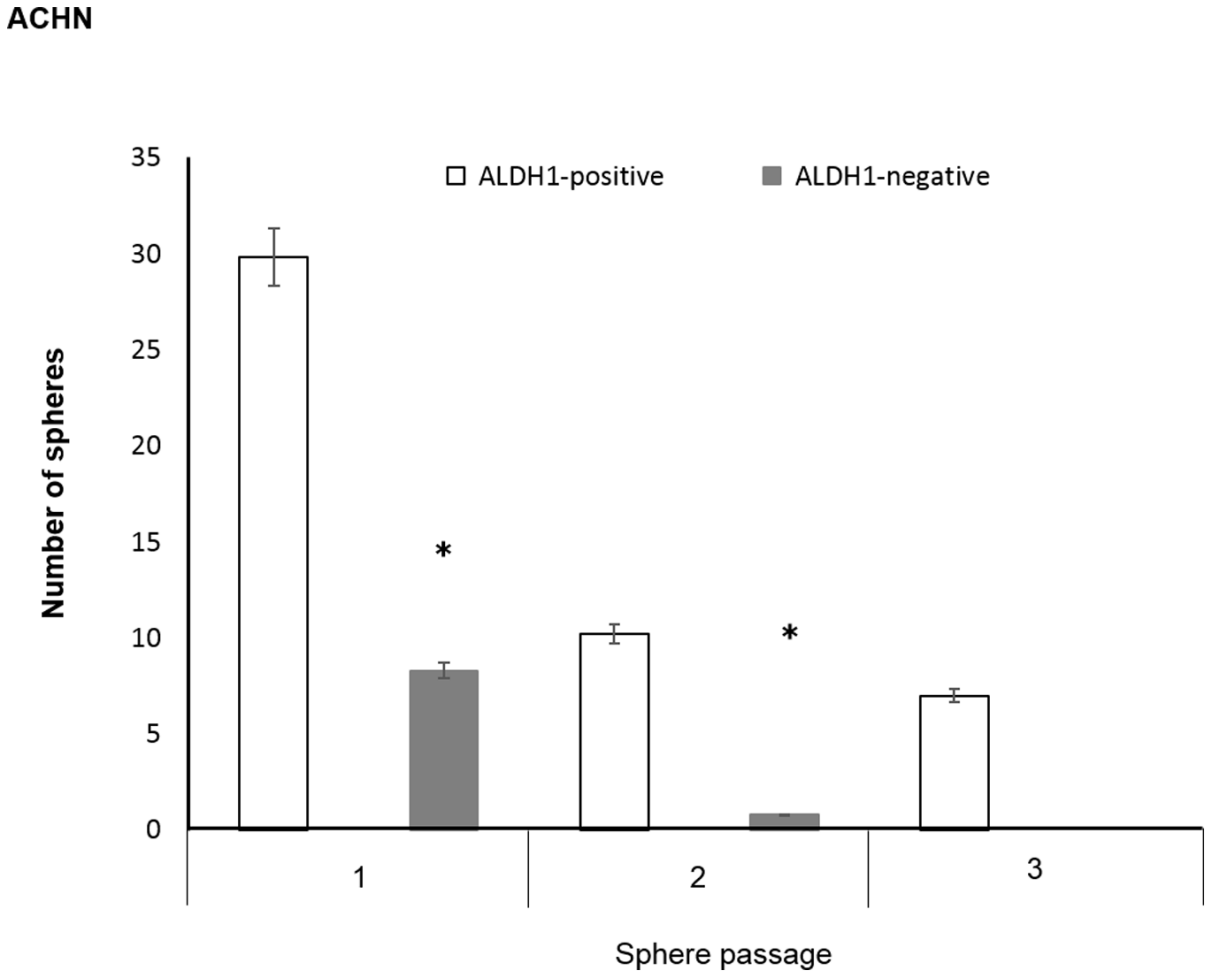
A self-renewal capacity between ALDH1-positive and ALDH1-negative ACHN cells. The sphere forming ability of ALDH1-positive cells in ACHN was maintained during the second and third passages (* *P*<0.0001).

The sphere forming ability of ALDH1-positive cells in both ACHN and KRC/Y was higher than that of ALDH1-negative cells. Moreover, ALDH1-positive cells in ACHN generated significantly larger sphere sizes than ALDH1-negative cells (Fig. 4D). Also, we found that single-dissociated sphere cells plated at a density of 4,000 cells per well gave rise to secondary and tertiary spheres within 1 week of seeding. Although the number of spheres was shown to decrease in the second and third passages compared to the first passage, the sphere forming ability of ALDH1-positive cells in ACHN was maintained during the second and third passages. On the other hand, ALDH1-negative cells formed a few secondary spheres (Fig. 5).

Tumor formation was observed in three of five and five of five mice injected with 10×10^3^ and 100×10^3^ ALDH1-positive cells, respectively, at 8 weeks. However, ALDH1-negative cell injection developed no visible tumors in all mice by this time (Table 2).

**Table 2:** Tumorigenicity of aldehyde dehydrogenase 1 (ALDH1)-positive and ALDH1-negative cells in ACHN.

	Injected cell number		
		1×10^4^	1×10^5^
**ACHN**	**ALDH1-positive**	3/5	5/5
	**ALDH1-negative**	0/5	0/5

### qRT-PCR in ALDH1-positive and ALDH1-negative ACHN Cells

**Figure 6:**
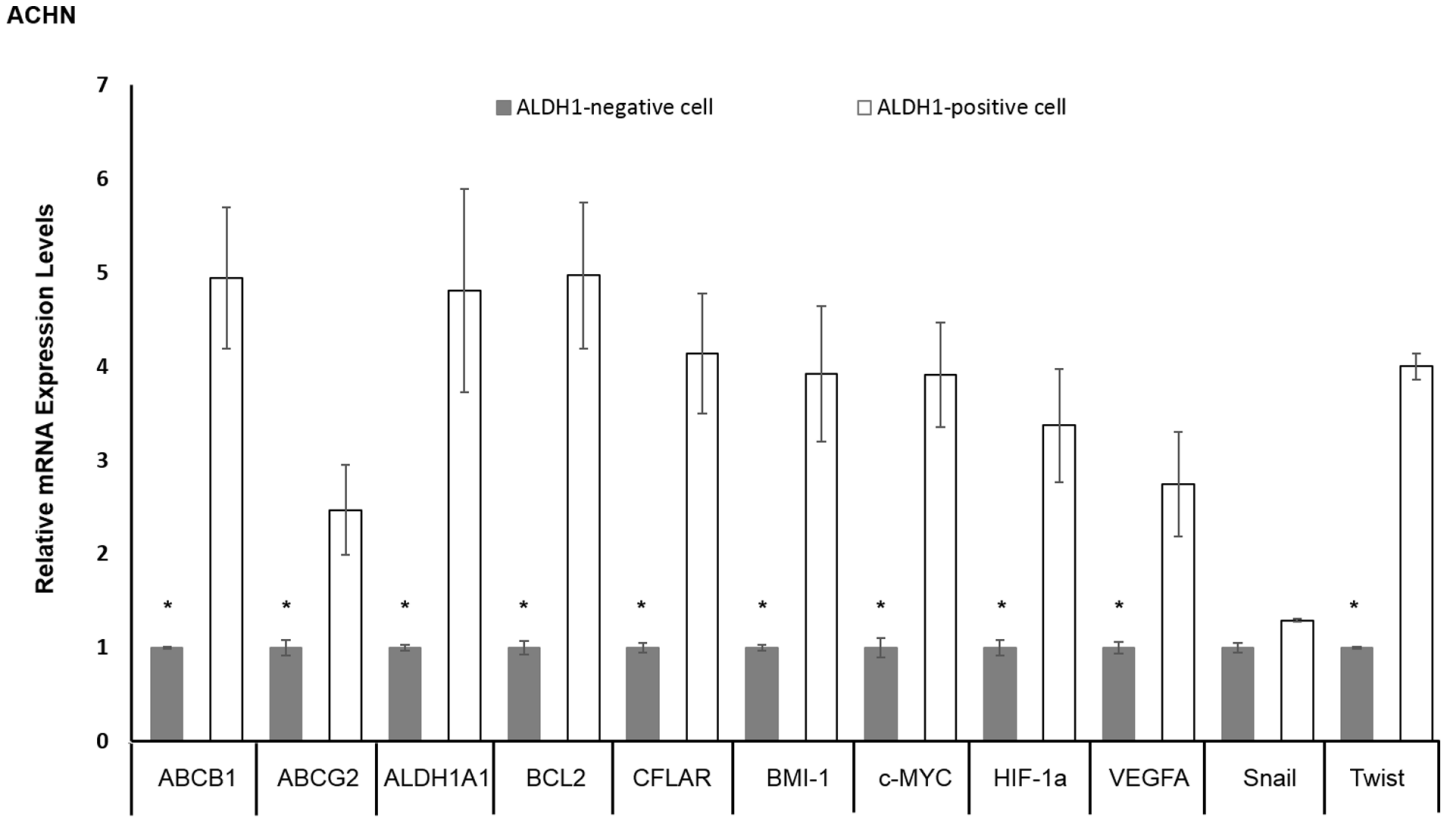
Quantification of mRNA expression of CSC-LC property-related genes in ALDH1-positive and ALDH1-negative ACHN cells by real-time PCR. ALDH1-positive cells showed significantly higher mRNA expression of ALDH1A1, transporter-related genes (ABCB1 and ABCG2), self-replication genes (BMI-1 and c-MYC), anti-apoptosis genes (BCL2 and CFLAR), hypoxia-related genes (HIF1α and VEGFA) and EMT-related genes (Twist) than ALDH1-negative cells in ACHN. However, there was no significant difference in mRNA expression of Snail between ALDH1-positive and ALDH1-negative cells. The experiments were repeated at least four times, and almost identical results were obtained.

We performed qRT-PCR analysis to compare CSC-LC property-related gene expression in ALDH1-positive and ALDH1-negative ACHN cells. ALDH1-positive cells expressed significantly higher levels of mRNA in all genes except Snail than ALDH1-negative cells. The levels of the increase were as follows: ABCB1, 4.9-fold; ABCG2, 2.5-fold, ALDH1A1, 4.8-fold; BCL2, 5.0-fold; CFLAR, 4.1-fold; BMI-1, 3.9-fold; c-MYC, 3.9-fold; HIF1α, 3.4-fold; VEGFA, 2.7-fold; Twist, 4.0-fold (Fig. 6).

## Discussion

Since the CSC concept was proposed to explain the heterogeneity of tumor cells, CSCs or CSC-LCs have been identified in many types of cancer. In general, CSCs possess both self-renewal and differentiation capabilities allowing CSC to partially recreate the cellular heterogeneity of the parental tumor. A number of studies have reported that the inability of conventional therapies to prevent recurrence or metastases is due to the presence of small subsets of resistant cells, namely CSCs [8], [30]. In recent years the SP technique has become one of the most widely used methods of isolating CSC-LCs. Since the detailed staining and measurement method of Goodell et al. was first introduced, many researchers have reported that SP cells are a subset of cells with higher grade malignancy, and CSC-LCs characteristics [15], [16], [31]. With regard to RCC, Addla et al. reported that SP cells accounted for 4–6% of total cancer cells. However the cellular characteristics of SP cells are not well understood [17].

In our present study we found that the SP fractions in ACHN and KRC/Y were 1.4% and 1.7%, respectively. There was no difference between KRC/Y SP and NSP cells in tumorigenicity, sphere forming ability, or in resistance to Sorafenib or IFNα, which are conventionally used to treat advanced RCC. These findings indicate that KRC/Y SP cells lack the characteristics of CSCs-LCs. In contrast, whereas there were no significant differences between ACHN SP and NSP cells in the in vitro cell growth or colony formation assays, SP cells did show a higher sphere forming ability, higher IFNα resistance and higher tumorigenicity in NOD/SCID mice than NSP cells, suggestive of cells with CSC-LC properties are included in ACHN SP cells. At present the SP approach is the most widely used method to identify CSC markers, however many researchers still question the relationship between SP cells and CSCs [32]–[34]. In addition, Ibrahim et al. studied the relationship between Hoechst staining concentration and incubation time and reported that Hoechst staining concentration had an effect on cell damage [35]. In our present study, in order to identify the SP cells in ACHN and KRC/Y we used Hoechst staining at a concentration of 5 µg/mL and 10 µg/mL, respectively. Hoechst staining is generally carried out at a concentration of 5 µg/mL, but in the present study we used a higher concentration in KRC/Y cells [36]. Thus, we cannot completely rule out the possibility that cellular damage due to Hoechst staining was responsible for the difference in biological characteristics observed between KRC/Y cells in vivo and in vitro in our current study.

Bussolati et al. previously reported in a human RCC cell line that CD105-positive cells represented a cell group with high clonogenicity and high tumorigenicity; however, our present study found that while KRC/Y SP cells contained about five times as many CD105-positive cells as KRC/Y NSP cells, there were no differences in CSC-LC properties between KRC/Y SP and NSP cells. In addition, CD105 expression was found in a few cells in the ACHN. Thus, our current results conflict with the findings of Bussolati et al., and suggest the possibility that CD105 may not be a universal CSC marker in RCC.

Many recent studies have reported that SP cells show a higher expression of ABC transporters, especially ABCG2, than NSP cells in many solid tumors and cell lines, and that this may play a role in drug efflux and drug resistance. The expression of drug transporters via ABCG2 is an important marker in the identification and analysis of SP cells [19], [20], [31], [37]. In our present study, we observed no difference in ABCG2 expression at the mRNA level between SP and NSP cells in either of the two RCC cell lines studied. However, in the past few years several studies have reported that SP cells express other transporters, such as ABCB1 and ABCB5, in addition to ABCG2 [38], [39]. Therefore, this result may be due to the expression of the other transporters in SP cells, or it may be because the functions of ABCB1 and ABCG2 were not reflected by mRNA expression of these genes. This point needs to be further studied.

Next, in order to study other CSC markers, we performed an Aldefluor assay. ALDH1 enzymatic activity has been recognized in recent years as a general marker of both normal stem cells and CSCs [40], [41]. ALDH1-positive cells have CSC-LC characteristics, such as the ability to self-replicate and to form tumors, so a number of researchers have used ALDH1 enzymatic activity as a CSC marker in many different types of cancer, including lung, liver, pancreas, prostate, bladder, breast and malignant melanoma [42]–[46]. It has also been reported in breast and several other cancers that high ALDH1 expression is closely associated with poor clinical prognosis [23]. Recently, sphere formation assays have been widely used to assess the self-renewal capacity of CSC-LCs. Our present study revealed that ACHN SP cells contain more ALDH1-positive cells than NSP cells and that not only ACHN but also KRC/Y ALDH1-positive cells had greater sphere forming ability. In order to elucidate our results, we performed subsequent generations of sphere forming assays. The self-renewal capacity of ALDH1-positive cells in ACHN, but not ALDH1-negative cells, was maintained for at least three generations. Furthermore, the tumorigenicity of ALDH1-positive cells was significantly higher than ALDH1-negative cells. These results indicate that ALDH1 could be a CSC marker in RCC. According to some recent reports, the VEGF-neutralizing antibody Bevacizumab, and anti-angiogenesis drugs such as Sorafenib and Sunitinib, which are VEGF receptor tyrosine kinase inhibitors, suppressed tumor proliferation, but at the same time promoted invasion and metastasis [47], [48]. Also, Conley et al. found in a breast cancer cell line that anti-angiogenesis therapy caused an increase in ALDH1-positive cells, indicating that these cells were associated with resistance to therapy [49]. The present study found that ALDH1-positive cells expanded chronologically under hypoxic conditions and after exposure to drugs. These findings indicate that ALDH1-positive cells are resistant to conventional therapies for RCC, and that they represent a cell fraction that can survive under hypoxic conditions and can replicate in adverse environments. Previous studies have reported that CSC-LCs have anti-apoptotic and drug resistant properties due to expression of anti-apoptosis genes such as BCL2 and CFLAR [50]. Moreover, recent studies have found that CSC-LCs occupy a hypoxic niche, that they can survive treatment with VEGFR2 inhibitors, and that they are involved in resistance to therapy [49], [51], [52]. Our real time PCR assays also found that self-replication markers such as BMI-1 and c-MYC were highly expressed in ALDH1-positive cells, along with a variety of drug efflux transporters. Moreover, anti-apoptosis genes such as BCL2 or CFLAR were also highly expressed in ALDH1-positive cells, along with HIF1α. These findings suggest that ALDH1-positive cells not only have anti-apoptotic effects, but also that they can survive under hypoxic conditions and could represent a cell population that is resistant to current conventional therapies. Our present study also found that ALDH1 expression was increased after drug treatment or exposure to hypoxia, which suggests the involvement of ALDH1-positive cells in drug resistance. Several recent reports have suggested that EMT also results in the acquisition of other properties involved in carcinoma progression, such as increased resistance to apoptosis and the acquisition of CSC-LC properties [52]. In our study, although Snail mRNA level was not significant different between ALDH1-positive and ALDH1-negative cells, Twist mRNA level was significantly increased in ALDH1-positive cells. These results suggest that ALDH1-positive cells may be related to EMT phenomenon. However, this finding needs to be further studied.

In conclusion, the results suggest that the ALDH1-positive cell population rather than SP cells shows CSC-LC properties in human RCC cells. Further studies are needed to determine the relationship between these findings and the clinical prognosis in RCC.

## References

[1] Kroeger N, Seligson DB, Klatte T, Rampersaud EN, Birkhauser FD, et al (2012) Clinical, molecular, and genetic correlates of lymphatic spread in clear cell renal cell carcinoma. Eur Urol 61: 888–895. 22269604 10.1016/j.eururo.2012.01.012

[2] Costa LJ, Drabkin HA (2007) Renal cell carcinoma: new developments in molecular biology and potential for targeted therapies. Oncologist 12: 1404–1415. 18165617 10.1634/theoncologist.12-12-1404

[3] Flanigan RC, Campbell SC, Clark JI, Picken MM (2003) Metastatic renal cell carcinoma. Curr Treat Options Oncol 4: 385–390. 12941198 10.1007/s11864-003-0039-2

[4] Motzer RJ, Hutson TE, Tomczak P, Michaelson MD, Bukowski RM, et al (2007) Sunitinib versus interferon alfa in metastatic renal-cell carcinoma. N Engl J Med 356: 115–124. 17215529 10.1056/NEJMoa065044

[5] Escudier B, Eisen T, Stadler WM, Szczylik C, Oudard S, et al (2007) Sorafenib in advanced clear-cell renal-cell carcinoma. N Engl J Med 356: 125–134. 17215530 10.1056/NEJMoa060655

[6] Motzer RJ, Escudier B, Oudard S, Hutson TE, Porta C, et al (2008) Efficacy of everolimus in advanced renal cell carcinoma: a double-blind, randomised, placebo-controlled phase III trial. Lancet 372: 449–456. 18653228 10.1016/S0140-6736(08)61039-9

[7] Bonnet D, Dick JE (1997) Human acute myeloid leukemia is organized as a hierarchy that originates from a primitive hematopoietic cell. Nat Med 3: 730–737. 9212098 10.1038/nm0797-730

[8] Nowell PC (1976) The clonal evolution of tumor cell populations. Science 194: 23–28. 959840 10.1126/science.959840

[9] Dalerba P, Cho RW, Clarke MF (2007) Cancer stem cells: models and concepts. Annu Rev Med 58: 267–284. 17002552 10.1146/annurev.med.58.062105.204854

[10] Visvader JE, Lindeman GJ (2008) Cancer stem cells in solid tumours: accumulating evidence and unresolved questions. Nat Rev Cancer 8: 755–768. 18784658 10.1038/nrc2499

[11] Bussolati B, Bruno S, Grange C, Ferrando U, Camussi G (2008) Identification of a tumor-initiating stem cell population in human renal carcinomas. FASEB J 22: 3696–3705. 18614581 10.1096/fj.08-102590

[12] Bussolati B, Brossa A, Camussi G (2011) Resident stem cells and renal carcinoma. Int J Nephrol 2011: 286985. 21647312 10.4061/2011/286985 PMC3106374

[13] Kim K, Ro JY, Kim S, Cho YM (2012) Expression of stem-cell markers OCT-4 and CD133: important prognostic factors in papillary renal cell carcinoma. Hum Pathol 43: 2109–2116. 22944295 10.1016/j.humpath.2012.05.006

[14] Kim K, Ihm H, Ro JY, Cho YM (2011) High-level expression of stem cell marker CD133 in clear cell renal cell carcinoma with favorable prognosis. Oncol Lett 2: 1095–1100. 22848273 10.3892/ol.2011.368 PMC3406563

[15] Hirschmann-Jax C, Foster AE, Wulf GG, Nuchtern JG, Jax TW, et al (2004) A distinct “side population” of cells with high drug efflux capacity in human tumor cells. Proc Natl Acad Sci U S A 101: 14228–14233. 15381773 10.1073/pnas.0400067101 PMC521140

[16] Hulspas R, Quesenberry PJ (2000) Characterization of neurosphere cell phenotypes by flow cytometry. Cytometry 40: 245–250. 10878568

[17] Addla SK, Brown MD, Hart CA, Ramani VA, Clarke NW (2008) Characterization of the Hoechst 33342 side population from normal and malignant human renal epithelial cells. Am J Physiol Renal Physiol 295: F680–687. 18614618 10.1152/ajprenal.90286.2008 PMC2536866

[18] Nishizawa S, Hirohashi Y, Torigoe T, Takahashi A, Tamura Y, et al (2012) HSP DNAJB8 controls tumor-initiating ability in renal cancer stem-like cells. Cancer Res 72: 2844–2854. 22552285 10.1158/0008-5472.CAN-11-3062

[19] Kondo T, Setoguchi T, Taga T (2004) Persistence of a small subpopulation of cancer stem-like cells in the C6 glioma cell line. Proc Natl Acad Sci U S A 101: 781–786. 14711994 10.1073/pnas.0307618100 PMC321758

[20] Loebinger MR, Giangreco A, Groot KR, Prichard L, Allen K, et al (2008) Squamous cell cancers contain a side population of stem-like cells that are made chemosensitive by ABC transporter blockade. Br J Cancer 98: 380–387. 18219291 10.1038/sj.bjc.6604185 PMC2361447

[21] Ikeda K, Saitoh S, Arase Y, Chayama K, Suzuki Y, et al (1999) Effect of interferon therapy on hepatocellular carcinogenesis in patients with chronic hepatitis type C: A long-term observation study of 1,643 patients using statistical bias correction with proportional hazard analysis. Hepatology 29: 1124–1130. 10094956 10.1002/hep.510290439

[22] Marcato P, Dean CA, Pan D, Araslanova R, Gillis M, et al (2011) Aldehyde dehydrogenase activity of breast cancer stem cells is primarily due to isoform ALDH1A3 and its expression is predictive of metastasis. Stem Cells 29: 32–45. 21280157 10.1002/stem.563

[23] Resetkova E, Reis-Filho JS, Jain RK, Mehta R, Thorat MA, et al (2010) Prognostic impact of ALDH1 in breast cancer: a story of stem cells and tumor microenvironment. Breast Cancer Res Treat 123: 97–108. 19911270 10.1007/s10549-009-0619-3

[24] Huang CP, Tsai MF, Chang TH, Tang WC, Chen SY, et al (2012) ALDH-positive lung cancer stem cells confer resistance to epidermal growth factor receptor tyrosine kinase inhibitors. Cancer Lett 328: 144–151. 22935675 10.1016/j.canlet.2012.08.021

[25] Ozbek E, Calik G, Otunctemur A, Aliskan T, Cakir S, et al (2012) Stem cell markers aldehyde dehydrogenase type 1 and nestin expressions in renal cell cancer. Arch Ital Urol Androl 84: 7–11. 22649953

[26] Yano H, Maruiwa M, Sugihara S, Kojiro M, Noda S, et al (1988) Establishment and characterization of a new human renal cell carcinoma cell line (KRC/Y). In Vitro Cell Dev Biol 24: 9–16. 3338971 10.1007/BF02623810

[27] Chiba T, Kita K, Zheng YW, Yokosuka O, Saisho H, et al (2006) Side population purified from hepatocellular carcinoma cells harbors cancer stem cell-like properties. Hepatology 44: 240–251. 16799977 10.1002/hep.21227

[28] Hisaka T, Yano H, Ogasawara S, Momosaki S, Nishida N, et al (2004) Interferon-alphaCon1 suppresses proliferation of liver cancer cell lines in vitro and in vivo. J Hepatol 41: 782–789. 15519651 10.1016/j.jhep.2004.07.012

[29] Lim YC, Oh SY, Cha YY, Kim SH, Jin X, et al (2011) Cancer stem cell traits in squamospheres derived from primary head and neck squamous cell carcinomas. Oral Oncol 47: 83–91. 21167769 10.1016/j.oraloncology.2010.11.011

[30] Reya T, Morrison SJ, Clarke MF, Weissman IL (2001) Stem cells, cancer, and cancer stem cells. Nature 414: 105–111. 11689955 10.1038/35102167

[31] Goodell MA, Brose K, Paradis G, Conner AS, Mulligan RC (1996) Isolation and functional properties of murine hematopoietic stem cells that are replicating in vivo. J Exp Med 183: 1797–1806. 8666936 10.1084/jem.183.4.1797 PMC2192511

[32] Burkert J, Otto WR, Wright NA (2008) Side populations of gastrointestinal cancers are not enriched in stem cells. J Pathol 214: 564–573. 18266310 10.1002/path.2307

[33] Takaishi S, Okumura T, Tu S, Wang SS, Shibata W, et al (2009) Identification of gastric cancer stem cells using the cell surface marker CD44. Stem Cells 27: 1006–1020. 19415765 10.1002/stem.30 PMC2746367

[34] Broadley KW, Hunn MK, Farrand KJ, Price KM, Grasso C, et al (2011) Side population is not necessary or sufficient for a cancer stem cell phenotype in glioblastoma multiforme. Stem Cells 29: 452–461. 21425408 10.1002/stem.582

[35] Ibrahim SF, Diercks AH, Petersen TW, van den Engh G (2007) Kinetic analyses as a critical parameter in defining the side population (SP) phenotype. Exp Cell Res 313: 1921–1926. 17428468 10.1016/j.yexcr.2007.02.025

[36] Ooka H, Kanda S, Okazaki H, Suzuki H, Mishima K, et al (2012) Characterization of side population (SP) cells in murine cochlear nucleus. Acta Otolaryngol 132: 693–701. 22667338 10.3109/00016489.2012.657358

[37] Goodell MA, Rosenzweig M, Kim H, Marks DF, DeMaria M, et al (1997) Dye efflux studies suggest that hematopoietic stem cells expressing low or undetectable levels of CD34 antigen exist in multiple species. Nat Med 3: 1337–1345. 9396603 10.1038/nm1297-1337

[38] Luo Y, Ellis LZ, Dallaglio K, Takeda M, Robinson WA, et al (2012) Side population cells from human melanoma tumors reveal diverse mechanisms for chemoresistance. J Invest Dermatol 132: 2440–2450. 22622430 10.1038/jid.2012.161 PMC3434242

[39] Smith PJ, Wiltshire M, Chappell SC, Cosentino L, Burns PA, et al (2012) Kinetic analysis of intracellular Hoechst 33342-DNA interactions by flow cytometry: Misinterpretation of side population status? Cytometry A 83: 161–169. 23136081 10.1002/cyto.a.22224

[40] Ma I, Allan AL (2011) The role of human aldehyde dehydrogenase in normal and cancer stem cells. Stem Cell Rev 7: 292–306. 21103958 10.1007/s12015-010-9208-4

[41] Marcato P, Dean CA, Giacomantonio CA, Lee PW (2011) Aldehyde dehydrogenase: its role as a cancer stem cell marker comes down to the specific isoform. Cell Cycle 10: 1378–1384. 21552008 10.4161/cc.10.9.15486

[42] Ma S, Chan KW, Lee TK, Tang KH, Wo JY, et al (2008) Aldehyde dehydrogenase discriminates the CD133 liver cancer stem cell populations. Mol Cancer Res 6: 1146–1153. 18644979 10.1158/1541-7786.MCR-08-0035

[43] Jiang F, Qiu Q, Khanna A, Todd NW, Deepak J, et al (2009) Aldehyde dehydrogenase 1 is a tumor stem cell-associated marker in lung cancer. Mol Cancer Res 7: 330–338. 19276181 10.1158/1541-7786.MCR-08-0393 PMC4255559

[44] Ginestier C, Hur MH, Charafe-Jauffret E, Monville F, Dutcher J, et al (2007) ALDH1 is a marker of normal and malignant human mammary stem cells and a predictor of poor clinical outcome. Cell Stem Cell 1: 555–567. 18371393 10.1016/j.stem.2007.08.014 PMC2423808

[45] Kim MP, Fleming JB, Wang H, Abbruzzese JL, Choi W, et al (2011) ALDH activity selectively defines an enhanced tumor-initiating cell population relative to CD133 expression in human pancreatic adenocarcinoma. PLoS One 6: e20636. 21695188 10.1371/journal.pone.0020636 PMC3113804

[46] van den Hoogen C, van der Horst G, Cheung H, Buijs JT, Lippitt JM, et al (2010) High aldehyde dehydrogenase activity identifies tumor-initiating and metastasis-initiating cells in human prostate cancer. Cancer Res 70: 5163–5173. 20516116 10.1158/0008-5472.CAN-09-3806

[47] Ebos JM, Lee CR, Cruz-Munoz W, Bjarnason GA, Christensen JG, et al (2009) Accelerated metastasis after short-term treatment with a potent inhibitor of tumor angiogenesis. Cancer Cell 15: 232–239. 19249681 10.1016/j.ccr.2009.01.021 PMC4540346

[48] Paez-Ribes M, Allen E, Hudock J, Takeda T, Okuyama H, et al (2009) Antiangiogenic therapy elicits malignant progression of tumors to increased local invasion and distant metastasis. Cancer Cell 15: 220–231. 19249680 10.1016/j.ccr.2009.01.027 PMC2874829

[49] Conley SJ, Gheordunescu E, Kakarala P, Newman B, Korkaya H, et al (2012) Antiangiogenic agents increase breast cancer stem cells via the generation of tumor hypoxia. Proc Natl Acad Sci U S A 109: 2784–2789. 22308314 10.1073/pnas.1018866109 PMC3286974

[50] Yajima T, Ochiai H, Uchiyama T, Takano N, Shibahara T, et al (2009) Resistance to cytotoxic chemotherapy-induced apoptosis in side population cells of human oral squamous cell carcinoma cell line Ho-1-N-1. Int J Oncol 35: 273–280. 19578740

[51] Borovski T, De Sousa EMF, Vermeulen L, Medema JP (2012) Cancer stem cell niche: the place to be. Cancer Res 71: 634–639. 10.1158/0008-5472.CAN-10-3220 21266356

[52] Heddleston JM, Li Z, McLendon RE, Hjelmeland AB, Rich JN (2009) The hypoxic microenvironment maintains glioblastoma stem cells and promotes reprogramming towards a cancer stem cell phenotype. Cell Cycle 8: 3274–3284. 19770585 10.4161/cc.8.20.9701 PMC2825672

